# Nomogram integrating BRAF V600E, serum biomarkers, and ultrasound features for malignancy risk stratification in C-TIRADS category 3 and 4 thyroid nodules

**DOI:** 10.3389/fmed.2026.1908160

**Published:** 2026-08-27

**Authors:** Yu Gao, Wenran Zhang, Jingmin Li, Jiaqing Dou

**Affiliations:** Department of Endocrinology, The Fourth Affiliated Hospital of Anhui Medical University, Hefei, China

**Keywords:** BRAF V600E mutation, C-TIRADS, nomogram, risk stratification, thyroid nodule, vascular endothelial growth factor

## Abstract

**Background:**

For patients with C-TIRADS 3 or 4 nodules who have undergone fine-needle aspiration cytology (FNAC), subsequent management decisions remain challenging, especially when cytological results are discordant with imaging findings or indeterminate. We built a nomogram incorporating BRAF V600E, serum biomarkers, and ultrasound features to provide an individualized risk estimate of malignancy for these nodules.

**Methods:**

We retrospectively included 300 patients with C-TIRADS 3 or 4 nodules. All nodules were confirmed by postoperative pathology or cytology. Among these, 76 were malignant and 224 benign. After running LASSO regression with 10-fold cross-validation and applying the 1-standard error rule to select core predictors, we built the nomogram using Firth-penalized logistic regression. Internal validity was checked using bootstrap resampling with 1,000 iterations while the model’s performance was evaluated using ROC curves, calibration (Hosmer–Lemeshow test, Brier score) and decision curve analysis (DCA). The additional predictive value beyond the C-TIRADS system was measured by the Net Reclassification Index (NRI) and the Integrated Discrimination Improvement (IDI).

**Results:**

Five independent predictors were finally selected: BRAF V600E mutation (OR = 36.455, 95% CI: 13.381–118.094), TgAb positivity (OR = 4.808, 95% CI: 2.289–10.486), microcalcifications (OR = 4.168, 95% CI: 1.880–9.424), aspect ratio > 1 (OR = 3.027, 95% CI: 1.285–7.115), and serum VEGF (per 100 pg/mL increase, OR = 3.798, 95% CI: 1.763–8.677). Each predictor was independently associated with malignancy (all *P* < 0.05). The area under the curve (AUC) of our nomogram reached 0.890 (95% CI: 0.845–0.935; bootstrap-corrected: 0.886), which was significantly higher than that of C-TIRADS alone (AUC = 0.746, *P* < 0.001). Reclassification analysis yielded a categorical NRI of 0.436 (95% CI: 0.316–0.551, *P* < 0.001) and an IDI of 0.263 (95% CI: 0.182–0.341, *P* < 0.001). Calibration was satisfactory: the Hosmer–Lemeshow *χ*^2^ gave 4.61 (*P* = 0.799), and the Brier score was 0.104. DCA demonstrated a higher net benefit across threshold probabilities ranging from 2 to 90%.

**Conclusion:**

The nomogram demonstrated good discriminative performance in C-TIRADS 3 or 4 nodules, supporting its utility as a post-FNAC decision aid.

## 1 Introduction

Nodules are the most common thyroid lesions. Over the past few years, their detection has increased steadily, largely due to the broad adoption of high-resolution ultrasound and expanded screening programs (1). Although only about 5–15% of nodules found on ultrasound are cancerous (2), accurately distinguishing benign from malignant lesions is essential. This distinction helps avoid overtreatment while ensuring timely intervention. Ultrasound serves as the first-line tool when a thyroid lump is suspected. Used together with the China Thyroid Imaging Reporting and Data System (C-TIRADS), which divides nodules into multiple risk categories based on standardized ultrasound criteria (3), it provides a unified framework for diagnosis and follow-up, with good clinical utility (4).

In clinical practice, C-TIRADS categories 3 and 4 account for the largest proportion of thyroid nodules. However, their malignancy risk varies widely, from 2 to 90% (3), and their optimal management is still debated. For category 3 lesions, current guidelines recommend regular follow-up. But one study based on multiple international grading systems reported malignancy rates of 8.7–10.7% in these low-risk nodules, exceeding the traditionally assumed rate (5). Given such a wide range, the C-TIRADS category by itself cannot guide individualized treatment decisions. Furthermore, benign and malignant nodules often share overlapping ultrasound features, and different observers may interpret the same sonograms differently (6, 7). FNAC is a widely used preoperative diagnostic technique for thyroid nodules (8). However, false-negative results may occur in some cases, particularly when sampling is inadequate or cytological interpretation is indeterminate. This limitation becomes particularly evident when cytology is indeterminate (Bethesda III/IV) or discordant with imaging findings; these are situations in which cytological information alone offers limited decision-making value.

Molecular and serological markers have recently gained attention as ancillary tools for thyroid nodule diagnosis. Among driver mutations in thyroid carcinoma, the BRAF V600E mutation ranks as the most frequent. Because of its high specificity, cytological diagnostic guidelines now recommend testing for it (9). However, a meta-analysis reported a sensitivity of only 40% for BRAF V600E testing (10). Hence, using it alone for screening is not reliable. Vascular endothelial growth factor (VEGF) helps regulate tumor angiogenesis, and multiple studies have found higher VEGF levels in thyroid cancer patients than in those with benign nodules (11, 12). Thyroid autoantibodies (such as TgAb and TPOAb) have also emerged as potential indicators. Their positivity is not only linked to autoimmune thyroid disease but may also raise the risk of thyroid cancer (13). In practice, however, no single marker performs adequately for clinical decision-making. Few prediction models have combined multiple molecular biomarkers with ultrasound features specifically for C-TIRADS 3 or 4 nodules.

We therefore built and internally validated a nomogram that integrates BRAF V600E mutation status, serum biomarkers, and sonographic features to estimate malignancy risk in C-TIRADS 3/4 nodules. This tool is specifically intended for use after FNAC, particularly in cytologically indeterminate or imaging-discordant cases, to provide a quantitative, individualized risk assessment that informs the clinical decision between surgery and active surveillance.

## 2 Materials and methods

### 2.1 Study subjects

This study was a retrospective cohort study. To ensure the quality and transparency of our reporting, we meticulously followed the TRIPOD-AI (Transparent Reporting of a Multivariable Prediction Model for Individual Prognosis Or Diagnosis—Artificial Intelligence) statement (14). We retrospectively enrolled 300 patients with C-TIRADS 3 or 4 nodules at the Fourth Affiliated Hospital of Anhui Medical University between October 2022 and January 2025, with one nodule included per patient (total 300 nodules) to avoid clustering effects from multiple nodules; the nodule with the highest C-TIRADS risk category was chosen as the representative nodule according to a pre-defined rule. If multiple nodules had the same C-TIRADS category, the nodule with the largest diameter was selected. Based on postoperative histopathology or cytology obtained via FNAC, patients were assigned to a malignant group (76 cases) or a benign group (224 cases). All malignant cases were confirmed by surgical histopathology. For the benign group, surgical histopathology was used when available; otherwise, classification relied on FNAC cytology showing Bethesda Category II (benign) plus at least 12 months of ultrasound follow-up. To minimize the risk of false-negative findings, we recommended a second FNAC or surgery if a nodule showed any sign of progression during follow-up (15): a ≥ 20% enlargement in at least two dimensions accompanied by an absolute increase of ≥2 mm, a volume increment exceeding 50%, or the appearance of new suspicious sonographic features (e.g., solid hypoechoic areas, aspect ratio >1, microcalcifications, or abnormal cervical lymph nodes).

**Figure 1:**
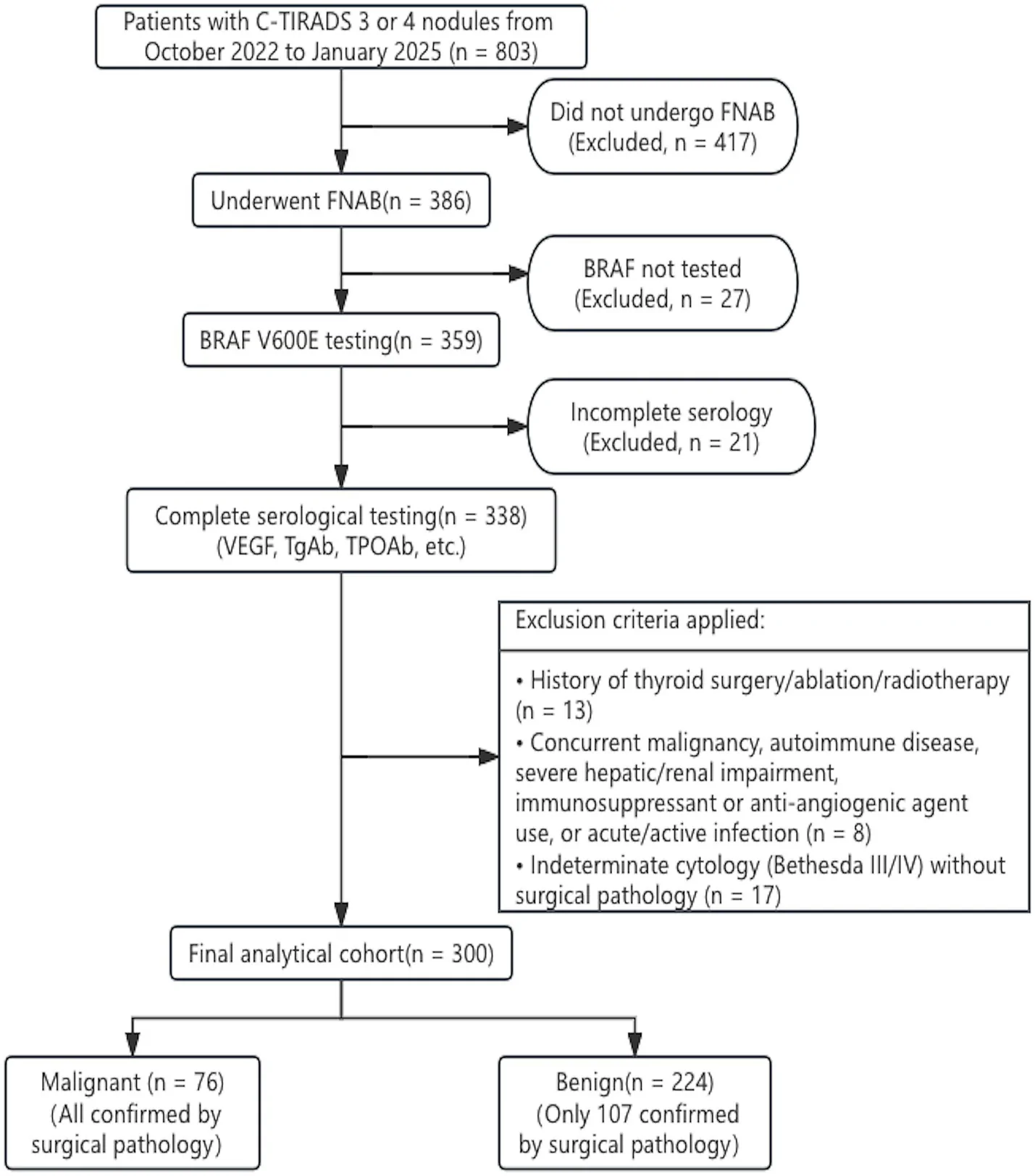
Participant flow diagram.

Inclusion Criteria: (1) C-TIRADS 3 or 4 classification of the thyroid nodule; (2) completion of serological testing, BRAF V600E genotyping, and neck ultrasound; (3) availability of full clinical or pathological information. Exclusion Criteria: (1) history of thyroid surgery, ablation therapy, or neck radiotherapy; (2) concurrent malignancy, other autoimmune disorders, or severe hepatic or renal impairment; (3) use of immunosuppressants or anti-angiogenic agents within 3 months before enrollment; (4) an acute or active infectious disease; (5) indeterminate cytology (Bethesda Category III or IV) without corresponding postoperative pathology; (6) missing values for any key predictor variable. These exclusion criteria were applied to minimize potential confounding from conditions that may independently affect VEGF, autoantibody levels, or inflammatory markers. The patient screening and selection process is illustrated in Figure 1. Our study protocol received approval from the Medical Ethics Committee of the Fourth Affiliated Hospital of Anhui Medical University (approval No. KYXM-202601-011). Informed consent was waived because the study was retrospective.

### 2.2 Sample size assessment

Sample size was estimated using the pmsampsize method recommended by Riley et al. (16). Based on the reported model performance (Cox-Snell *R*^2^ = 0.3988) from Fernández Alba et al. (17), considering all 19 candidate predictors included in the LASSO selection, and assuming a shrinkage factor of 0.9, the minimum required sample size was estimated as 533 patients with at least 135 events. Our study included 300 patients (76 events), falling below this conservative threshold. To mitigate the risk of overfitting associated with this sample size limitation, we employed LASSO regression with 10-fold cross-validation using the 1‑SE rule for predictor selection, and subsequently assessed model stability via bootstrap internal validation.

### 2.3 Data collection

We gathered clinical data retrospectively from the hospital’s electronic medical records. The data included patient age, sex, BMI, and results of routine laboratory tests. The laboratory tests included the following: thyroid peroxidase antibodies (TPOAb: positivity defined as >34.00 IU/mL; normal range 0.00–34.00 IU/mL), thyroglobulin antibodies (TgAb: positivity defined as >115.00 IU/mL; normal range 0.00–115.00 IU/mL), serum vascular endothelial growth factor (VEGF, units: pg/mL), neutrophil count (NEU), lymphocyte count (LYM), monocyte count (MON), and platelet count (PLT). The neutrophil-to-lymphocyte ratio (NLR), platelet-to-lymphocyte ratio (PLR), and monocyte-to-lymphocyte ratio (MLR) were calculated. We measured complete blood count parameters using a fully automated hematology analyzer (Sysmex XN-9000); TPOAb and TgAb were quantified by chemiluminescent immunoassay (Roche cobas e801); VEGF levels were measured by an ELISA kit (Jiangsu Meimian Industrial Co., Ltd.) in line with the supplier’s protocol, with results given in pg/mL.

### 2.4 Ultrasound examination and feature assessment

For ultrasound imaging, we employed a Samsung HERA W9 color Doppler system, which came with a high-frequency linear array transducer (5–12 MHz). An experienced radiologist performed real-time ultrasound scanning and provided an initial nodule grade. For model development, two senior radiologists (each with >10 years of experience) independently reviewed the stored images, blinded to pathology, BRAF V600E mutation status, and serological data. In cases of disagreement, a third senior sonographer (≥15 years of experience) served as the arbitrator, who was also blinded to these data. The following features were recorded: maximum nodule diameter, echogenicity, aspect ratio, shape, border clarity, composition, microcalcifications, blood flow signals, and enlarged cervical lymph nodes. Interobserver agreement was evaluated using Cohen’s Kappa coefficient for these features, with values >0.80 considered good. Interobserver agreement between the two radiologists was good for all ultrasound features, with Kappa values ranging from 0.80 to 0.92 (Supplementary Table S1).

### 2.5 FNAC

Ultrasound-guided FNAC was performed by an experienced senior endocrinologist. Under ultrasound guidance, a fine needle (23G) was used to repeatedly puncture the target nodule and perform negative-pressure aspiration to obtain an adequate number of cells. A portion of the aspirated specimen was prepared as a smear and stained; cytological grading and reporting were then conducted by a pathologist with extensive experience in thyroid cytopathology. Cytopathologists were blinded to BRAF V600E mutation status and ultrasound findings during assessment.

### 2.6 BRAF V600E mutation testing

Genomic DNA was isolated from the specimen by means of a micro-tissue DNA extraction kit, and a SLAN-96S real-time qPCR system was used to detect the BRAF V600E mutation status. A positive mutation was defined as a FAM channel signal Ct value < 38 and ΔCt < 9 (ΔCt = mutation Ct value − internal control Ct value). Each batch of testing included positive, negative, and blank controls to ensure the accuracy of the results. Histopathologists were similarly blinded to BRAF V600E mutation status and ultrasound findings.

### 2.7 Statistical analysis

We assessed normality using the Shapiro–Wilk test and *Q*–*Q* plots. Normally distributed variables were expressed as mean ± SD, with comparisons performed by independent *t*-tests. Non-normally distributed variables were shown as median (IQR) and groups were compared using the Mann–Whitney *U* test. Categorical variables were presented as frequencies (percentages), and compared using chi-square or Fisher’s exact test. Supplementary Table S2 provides details on variable coding.

Because a one-unit change in the raw scale (1 pg/mL) has limited clinical interpretability, we divided all VEGF measurements by 100 and used “per 100 pg/mL” as the unit for regression analyses and restricted cubic spline tests. We fit a restricted cubic spline (knots at the 10th, 50th, and 90th percentiles) to assess the dose–response pattern. When the nonlinear component gave *P* > 0.05, VEGF was treated as a linear continuous predictor.

LASSO regression with 10-fold cross-validation and the 1-SE rule (with random seed set to 123 for reproducibility) was applied to select predictors from the 19 candidate variables (Supplementary Table S2). The variance inflation factor (VIF) was used to check for multicollinearity, and a VIF below 5 indicated no serious collinearity. Because the number of malignant events was small and the BRAF V600E mutation was unevenly distributed between the two groups, standard logistic regression was prone to sparse-data bias. Consequently, the final model was fitted using Firth-penalized logistic regression.

Discrimination was quantified by the area under the ROC curve (AUC). Calibration was evaluated using calibration curves, the Hosmer–Lemeshow test (*P* > 0.05 considered acceptable), and the Brier score. Net clinical benefit across a range of thresholds was estimated by decision curve analysis (DCA). As we had no external validation set, we performed internal validation via bootstrap resampling (1,000 iterations, random seed = 123), yielding optimism-corrected AUC values.

For comparison with the C-TIRADS system, C-TIRADS categories were treated as dummy variables with category 3 (C-TIRADS 3) as the reference group, and a univariate logistic regression model was built accordingly. Differences in AUC were tested using the DeLong method. We calculated the categorical net reclassification index (NRI) and integrated discrimination improvement (IDI), which reflect the added predictive value of our nomogram. Coefficient estimates from conventional logistic regression were also compared with those from the Firth-penalized model to check robustness.

All analyses were carried out using R version 4.5.2 and SPSS 27.0. Statistical significance was set at two-sided *P* < 0.05.

## 3 Results

To minimize the potential impact of false-negative FNAC results, 117 nodules in the benign group—for which no postoperative pathology was available and which were diagnosed as Bethesda Category II based solely on FNAC cytology—underwent ultrasound follow-up for at least 12 months. The follow-up showed that 112 nodules (95.7%) stayed stable or decreased in size, whereas 5 nodules (4.3%) showed radiographic progression. Among the 5 progressing nodules, 4 underwent a second FNAC, which again yielded a Bethesda Category II (benign) result; the remaining 1 nodule with progression was surgically excised, and the postoperative pathology was also benign. Therefore, none of the nodules in this subgroup were reclassified as malignant after follow-up.

### 3.1 Baseline characteristics of the study population

Of the 300 enrolled patients, 76 (25.3%) had malignant nodules and 224 (74.7%) had benign nodules. Among the 76 malignant nodules, 74 (97.4%) were papillary thyroid carcinoma and 2 (2.6%) were follicular thyroid carcinoma. No other histological subtypes were identified in this cohort. The BRAF V600E mutation was detected in 47.4% of malignant cases, compared to only 2.2% of benign ones (*P* < 0.001). Serum VEGF levels were likewise elevated in the malignant group (median 241.22 pg/mL) relative to the benign group (214.10 pg/mL, *P* < 0.001). Malignant nodules also showed higher rates of autoantibody positivity: TgAb (35.5% vs. 23.7%, *P* = 0.043) and TPOAb (39.5% vs. 25.9%, *P* = 0.025). Regarding ultrasound features, malignant nodules more often exhibited a maximum diameter >1 cm, an aspect ratio >1, hypoechoic appearance, microcalcifications, increased internal or peripheral blood flow, and enlarged cervical lymph nodes (all *P* < 0.05). Sex, age, BMI, NLR, PLR, and MLR did not differ significantly between the two groups (*P* > 0.05). Table 1 provides a detailed breakdown.

**Table 1:** Baseline characteristics of the 300 patients.

Variables	Benign nodule (*n* = 224)	Malignant nodule (*n* = 76)	*Z*/*χ*^2^	*P*
Age (years)	55 (48.00, 60.00)	52.5 (40.25, 59.75)	−1.472	0.141
Gender, *n* (%)			0.300	0.584
Female	186 (83.0)	61 (80.3)		
Male	38 (17.0)	15 (19.7)		
BMI (kg/m^2^)	23.99 (21.79, 26.10)	24.63 (22.20, 27.40)	−1.147	0.251
BRAF V600E mutation, *n* (%)			97.983	<0.001
Wild-type	219 (97.8)	40 (52.6)		
Mutated	5 (2.2)	36 (47.4)		
VEGF (pg/mL)	214.10 (179.96, 247.80)	241.22 (202.83, 277.02)	−4.117	<0.001
NLR	1.72 (1.31, 2.28)	1.71 (1.29, 2.24)	−0.115	0.909
PLR	109.98 (83.55, 140.32)	101.46 (84.17, 129.84)	−0.708	0.479
MLR	0.17 (0.14, 0.21)	0.16 (0.14, 0.20)	−0.097	0.922
TgAb positivity, *n* (%)	53 (23.7)	27 (35.5)	4.086	0.043
TPOAb positivity, *n* (%)	58 (25.9)	30 (39.5)	5.049	0.025
Ultrasound features, *n* (%)				
Maximum diameter			8.120	0.004
≤1 cm	46 (20.5)	28 (36.8)		
>1 cm	178 (79.5)	48 (63.2)		
Aspect ratio			28.875	<0.001
≤1	203 (90.6)	49 (64.5)		
>1	21 (9.4)	27 (35.5)		
Echogenicity			11.431	<0.001
Non-hypoechoic	89 (39.7)	14 (18.4)		
Hypoechoic	135 (60.3)	62 (81.6)		
Shape			12.690	<0.001
Regular	196 (87.5)	53 (69.7)		
Irregular	28 (12.5)	23 (30.3)		
Border clarity			8.059	0.005
Clear	196 (87.5)	56 (73.7)		
Obscure	28 (12.5)	20 (26.3)		
Composition			6.688	0.010
Non-solid	51 (22.8)	7 (9.2)		
Solid	173 (77.2)	69 (90.8)		
Microcalcifications			23.020	<0.001
No	193 (86.2)	46 (60.5)		
Yes	31 (13.8)	30 (39.5)		
Blood flow signal			6.397	<0.001
No	191 (85.3)	55 (72.4)		
Yes	33 (14.7)	21 (27.6)		
Enlarged cervical lymph nodes			9.970	0.002
Invisible	179 (79.9)	47 (61.8)		
Visible	45 (20.1)	29 (38.2)		

Abbreviations: BMI, body mass index; VEGF, vascular endothelial growth factor; NLR, neutrophil-to-lymphocyte ratio; PLR, platelet-to-lymphocyte ratio; MLR, monocyte-to-lymphocyte ratio; TgAb, thyroglobulin antibody; TPOAb, thyroid peroxidase antibody.

### 3.2 Nonlinear relationship of serum VEGF

To model the dose–response pattern, we performed a nonlinear test with a restricted cubic spline (three knots at the 10th, 50th, and 90th percentiles) (Supplementary Figure S1). The overall association between VEGF and malignancy risk was significant (*χ*^2^ = 20.89, *P* < 0.001), whereas the nonlinear component was not (*P* = 0.135), indicating a linear relationship.

### 3.3 Predictor variable selection and collinearity diagnosis

**Figure 2:**
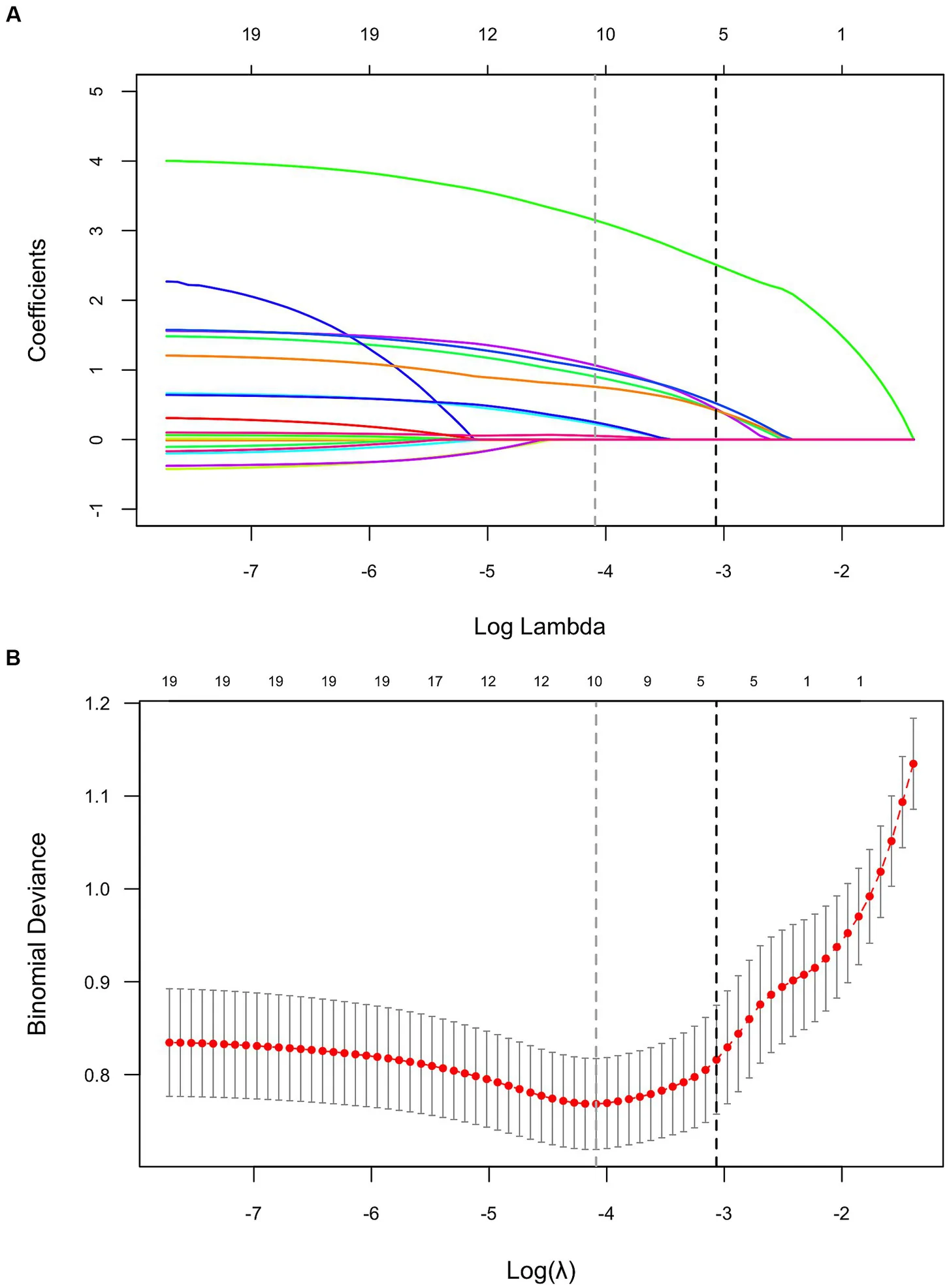
Variable selection using LASSO regression. We analyzed 19 candidate variables using LASSO regression with 10-fold cross-validation and the 1-SE criterion. Panel **(A)** displays the coefficient paths: Each line shows how a variable’s coefficient shrinks as the penalty parameter *λ* increases. Panel **(B)** plots the cross-validation binomial deviance against log(*λ*). The black vertical dashed line marks the optimal *λ* selected by the 1-SE rule, corresponding to log(*λ*) ≈ −3 (*λ* ≈ 0.0466). This selection yielded five predictors with non-zero coefficients: BRAF V600E mutation, TgAb positivity, microcalcifications, aspect ratio >1, and serum VEGF.

We screened 19 candidate predictors with LASSO regression (Figure 2). With 10-fold cross-validation and the 1-SE rule (*λ* = 0.0466), five predictors with non-zero coefficients emerged: BRAF V600E mutation, TgAb positivity, microcalcifications, aspect ratio > 1, and serum VEGF. All VIFs were under 2 (max 1.187), indicating no serious multicollinearity and supporting stable coefficient estimates (Supplementary Table S3).

### 3.4 Multivariate logistic regression analysis and nomogram construction

Based on the five LASSO-selected variables, we built a final predictive model using Firth-penalized logistic regression (Table 2). All five variables were independent predictors (all *P* < 0.05). The BRAF V600E mutation had the strongest effect (OR = 36.455, 95% CI: 13.381–118.094), followed by TgAb positivity (OR = 4.808, 95% CI: 2.289–10.486), microcalcifications (OR = 4.168, 95% CI: 1.880–9.424), aspect ratio > 1 (OR = 3.027, 95% CI: 1.285–7.115), and serum VEGF (per 100 pg/mL increase) (OR = 3.798, 95% CI: 1.763–8.677).

**Table 2:** Multivariable Firth-penalized logistic regression results.

Variables	*β*	S.E.	OR (95% CI)	*P*
BRAF V600E mutation	3.596	0.566	36.455 (13.381–118.094)	<0.001
TgAb positivity	1.570	0.391	4.808 (2.289–10.486)	<0.001
Aspect ratio >1	1.108	0.444	3.027 (1.285–7.115)	0.012
Microcalcifications	1.427	0.413	4.168 (1.880–9.424)	<0.001
VEGF (per 100 pg/mL)	1.335	0.364	3.798 (1.763–8.677)	0.002
Constant	−3.406	0.399	0.033 (0.014–0.069)	<0.001

OR, odds ratio; CI, confidence interval. We divided VEGF by 100 before modeling to make the effect size clinically meaningful. All five predictors remained statistically significant.

**Figure 3:**
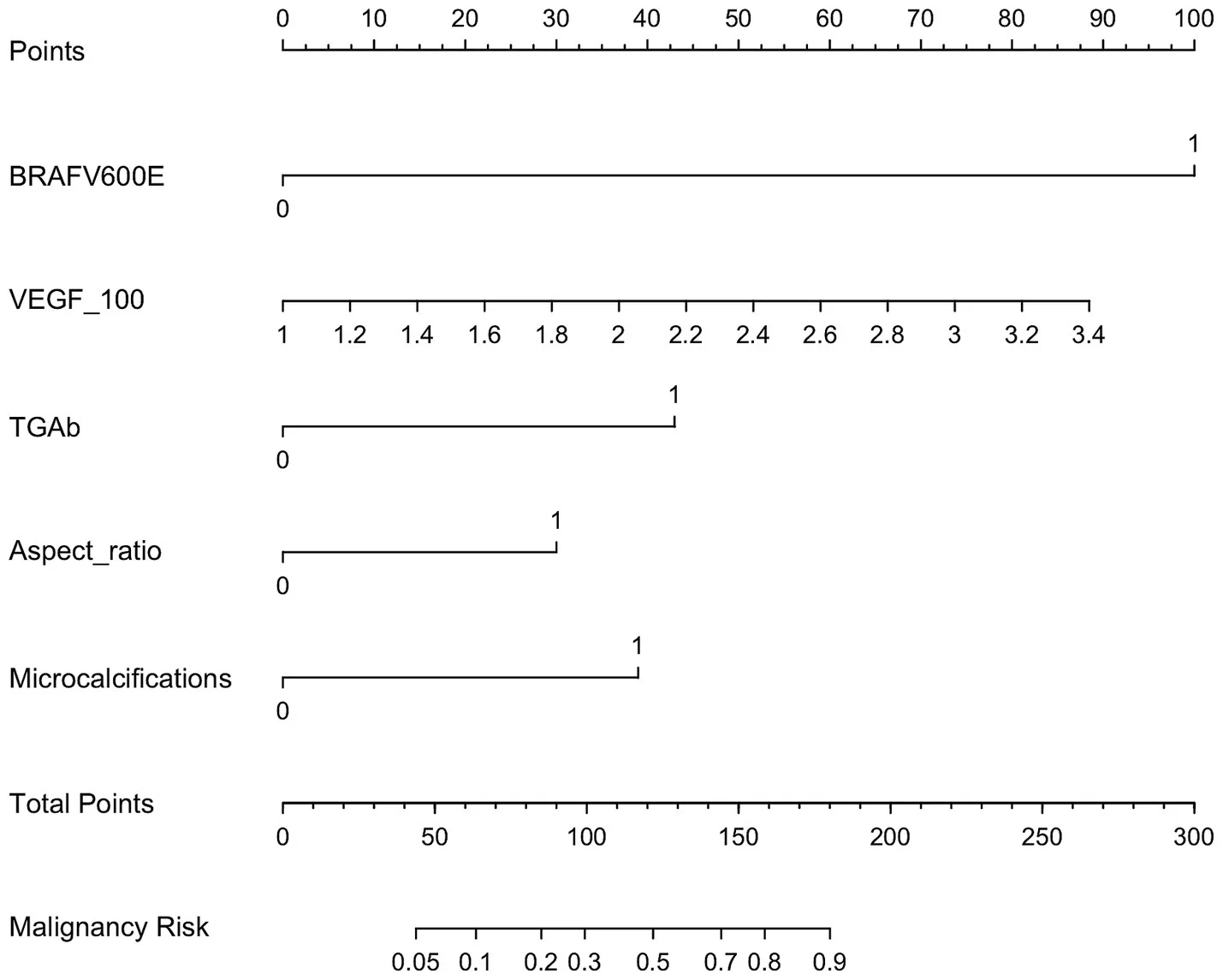
Nomogram for estimating malignancy risk in C-TIRADS 3 or 4 nodules. The nomogram integrates five predictors: BRAF V600E mutation, TgAb positivity, microcalcifications, aspect ratio >1, and serum VEGF (per 100 pg/mL). To obtain a predicted probability, locate the patient’s value on each predictor axis, draw a vertical line upward to the “Points” scale, and record the points. Sum all points, find the total on the “Total Points” axis, and then draw a vertical line downward to the “Risk of Malignancy” axis. For serum VEGF, divide the raw value (pg/mL) by 100 before using the nomogram.

A nomogram based on these five predictors is presented in Figure 3. Add up the points for each predictor to get the malignancy risk estimate from the nomogram.

### 3.5 Model evaluation and validation

**Figure 4:**
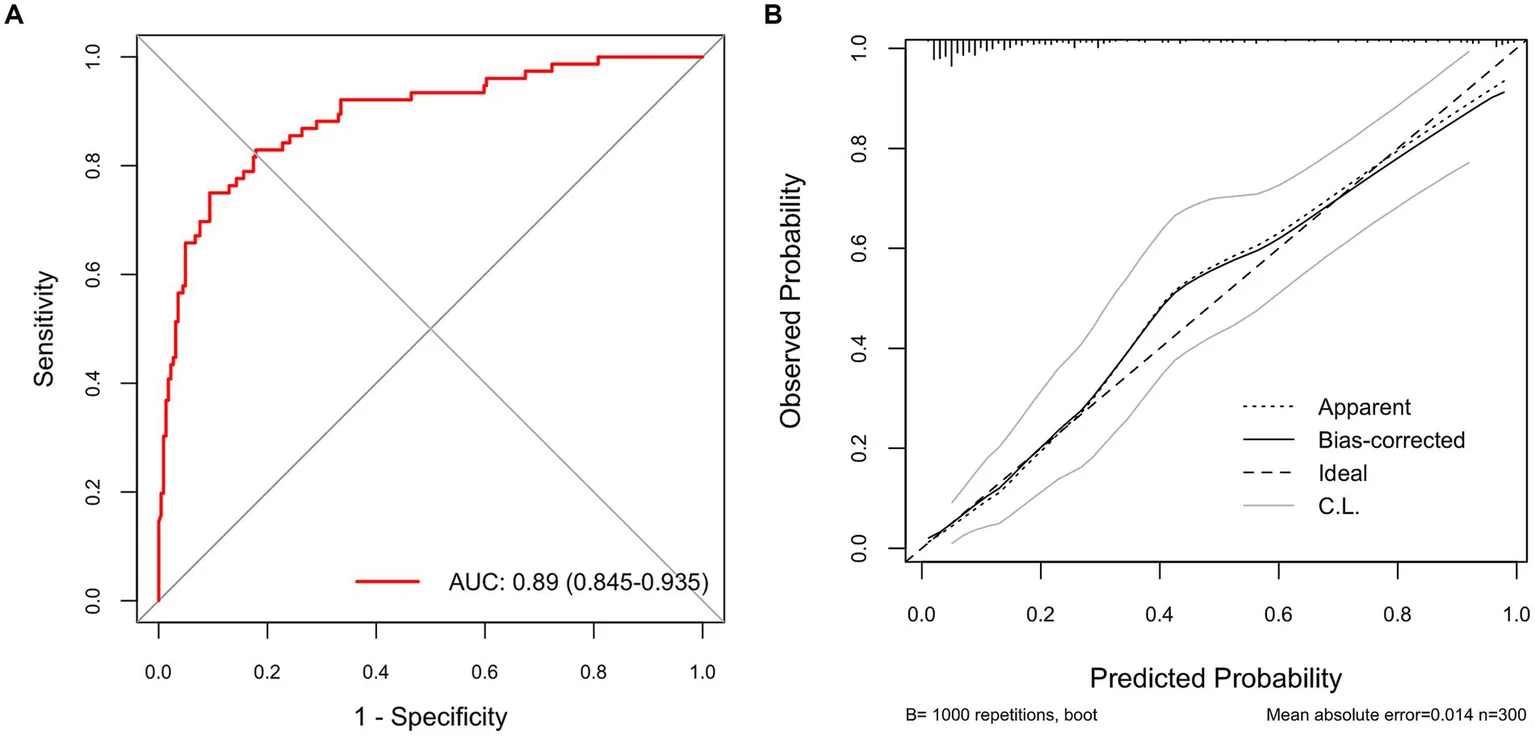
Discrimination and calibration of the nomogram. **(A)** ROC curve: The nomogram achieved an AUC of 0.890 (95% CI: 0.845–0.935). The solid diagonal line is the reference (AUC = 0.5). **(B)** Calibration plot: Predicted probabilities from the nomogram are plotted against observed malignancy proportions. The dashed diagonal line represents perfect calibration; the solid line (bias‑corrected) shows the model’s actual performance. The Hosmer–Lemeshow test (*χ*^2^ = 4.61, *p* = 0.799) and Brier score (0.104) confirmed good calibration.

Our nomogram achieved an AUC of 0.890 (95% CI: 0.845–0.935). Bootstrap internal validation with 1,000 resamples gave a corrected AUC of 0.886 and an optimism bias of 0.004, suggesting low overfitting and good internal stability (Figure 4A).

The Hosmer–Lemeshow test yielded a *χ*^2^ of 4.61 (*P* = 0.799), and the Brier score was 0.104. The calibration plot (Figure 4B) showed that predicted probabilities closely matched observed outcomes. These results indicate excellent calibration with no systematic bias between predicted and actual malignancy rates.

### 3.6 Sensitivity analysis for verification bias

To assess the potential impact of verification bias, we performed a sensitivity analysis restricted to the 183 patients with definitive surgical histopathology (107 benign and 76 malignant). In this gold-standard subgroup, the nomogram achieved an AUC of 0.897 (95% CI: 0.850–0.944), closely approximating the full-cohort AUC of 0.890 (95% CI: 0.845–0.935). Calibration remained satisfactory (Hosmer–Lemeshow *χ*^2^ = 7.12, *P* = 0.524; Brier score = 0.127). These findings suggest that the inclusion of FNAC-based benign diagnoses did not materially bias the model’s performance.

### 3.7 Model comparison

In our cohort, the C-TIRADS system achieved an AUC of 0.746 (95% CI: 0.687–0.806). The DeLong test indicated that our nomogram had a significantly higher AUC (*P* < 0.001); see Table 3 for details. Reclassification analysis yielded an NRI of 0.436 (95% CI: 0.316–0.551, *P* < 0.001) and an IDI of 0.263 (95% CI: 0.182–0.341, *P* < 0.001), indicating that the nomogram improved both reclassification and overall discrimination over C-TIRADS.

**Table 3:** Comparison of predictive performance.

Model	AUC (95% CI)	Sensitivity	Specificity	PPV	NPV	Accuracy	*P* value
Nomogram	0.890 (0.845–0.935)	0.750	0.906	0.731	0.914	0.867	Reference
C-TIRADS	0.746 (0.687–0.806)	0.724	0.750	0.495	0.889	0.743	<0.001^a^

a DeLong test *P* value comparing the AUC of C-TIRADS with that of the nomogram. AUC, area under the ROC curve; PPV, positive predictive value; NPV, negative predictive value.

### 3.8 Decision curve analysis

**Figure 5:**
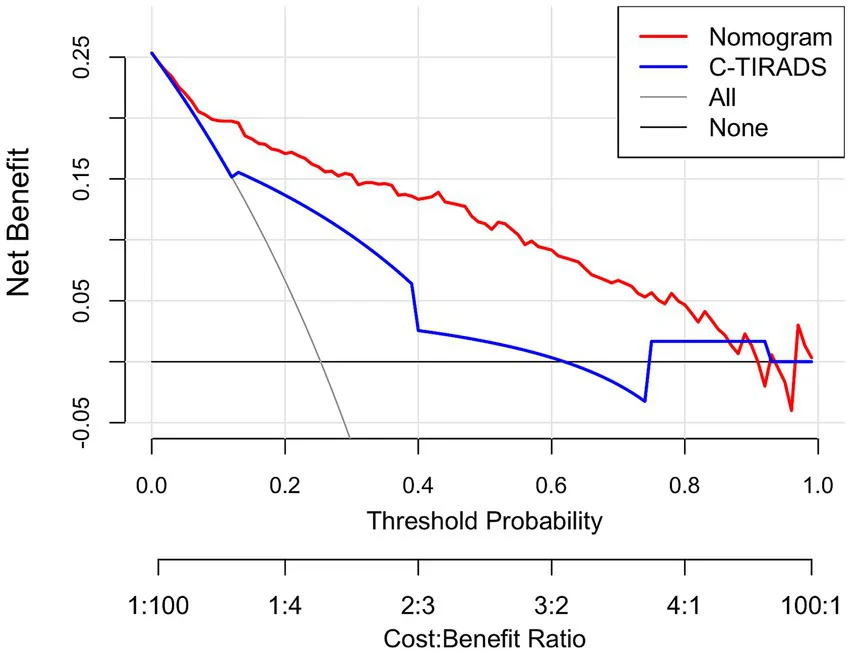
Decision curve analysis (DCA). DCA compares the clinical net benefit of the nomogram, C-TIRADS classification alone, “intervene on all,” and “intervene on none” across different threshold probabilities. The nomogram’s net benefit curve (red solid line) stays above the others for thresholds between approximately 2 and 90%, indicating that using the nomogram improves clinical decisions in that range.

Figure 5 presents the DCA. Across threshold probabilities from about 2 to 90%, the nomogram’s net benefit curve stayed above those of C-TIRADS, the “treat all” strategy, and the “treat none” strategy. Hence, the nomogram’s net clinical benefit is superior across the 2–90% threshold range.

### 3.9 Robustness analysis

We compared coefficient estimates from standard logistic regression and Firth-penalized regression (Table 4). The two methods gave the same coefficient directions for every predictor, and all five variables remained statistically significant in both models (all *P* < 0.05). The Firth-penalized estimates closely matched those from standard logistic regression, confirming coefficient robustness.

**Table 4:** Robustness of coefficient estimates.

Predictor	Standard logistic regression		Firth-penalized logistic regression	
	OR (95% CI)	*P*	OR (95% CI)	*P*
BRAF V600E mutation	43.467 (15.296–149.610)	<0.001	36.455 (13.381–118.094)	<0.001
TgAb positivity	5.058 (2.370–11.242)	<0.001	4.808 (2.289–10.486)	<0.001
Aspect ratio >1	3.102 (1.289–7.444)	0.011	3.027 (1.285–7.115)	0.012
Microcalcifications	4.349 (1.926–10.026)	<0.001	4.168 (1.880–9.424)	<0.001
VEGF (per 100 pg/mL)	4.017 (1.832–9.361)	0.001	3.798 (1.763–8.677)	0.001

OR, odds ratio; CI, confidence interval. Standard logistic regression used maximum likelihood; Firth-penalized logistic regression accounted for sparse data. Effect directions and significance were consistent across both methods, confirming robustness.

## 4 Discussion

The clinical challenge of C-TIRADS 3/4 nodules lies not only in the wide risk spectrum of ultrasound features, but also in the limited diagnostic accuracy of cytology when results are indeterminate or discordant with imaging, particularly after FNAC. We constructed a nomogram incorporating the BRAF V600E mutation, serum VEGF, TgAb, and two key ultrasound features (microcalcifications and an aspect ratio >1) to refine risk stratification in this setting. The nomogram distinguished benign from malignant lesions accurately (AUC = 0.890, 95% CI: 0.845–0.935) and showed good calibration (Hosmer–Lemeshow *χ*^2^ = 4.61, *P* = 0.799). In head-to-head comparison with C-TIRADS, the nomogram achieved a higher AUC (0.890 vs. 0.746, *P* < 0.001), better reclassification (NRI = 0.436, IDI = 0.263), and greater net clinical benefit across threshold probabilities of 2–90%. Overall, adding molecular and serological markers to ultrasound appears beneficial for managing thyroid nodules.

In our cohort, among the five independent predictors, the BRAF V600E mutation carried the strongest weight, with an OR of 36.455. This mutation keeps BRAF kinase constantly active, thereby upregulating MAPK signaling (18, 19). Several cellular changes occur, such as abnormal proliferation of thyroid follicular epithelial cells, failure to differentiate properly, and escape from programmed cell death. Each of these changes contributes to tumor initiation and progression. In East Asian populations, the BRAF V600E mutation rate in thyroid cancer has been reported to exceed 70% (20). In contrast, we detected it in only 47.4% of our malignant group. One possible reason for this discrepancy is that earlier studies included nodules from all categories, whereas we restricted to grades 3 and 4. The BRAF V600E mutation is a well-known marker of aggressive tumor behavior and high-risk ultrasound features; accordingly, its frequency increases progressively with higher nodule categories (21, 22).

Positive serum TPOAb or TgAb levels usually indicate chronic lymphocytic infiltration of the thyroid. In this autoimmune inflammatory setting, oxidative stress and DNA damage occur, which further leads to genetic instability and malignant transformation of follicular epithelial cells (23, 24). Our data showed that both TPOAb and TgAb positivity were more common in malignant than benign nodules, mirroring the results reported by Zhang et al. (25). However, during LASSO screening with the 1-SE rule, the coefficient for TPOAb shrank to zero and was dropped from the final model, unlike TgAb. This finding implies that when both markers enter the analysis, TgAb may offer more reliable independent predictive value.

Thyroid tumor cells may synthesize thyroglobulin carrying abnormal antigenic epitopes. This in turn provokes a specific immune response, raising TgAb levels in the bloodstream (13, 26). TgAb positivity also associates with aggressive cancer features, such as lymph node metastasis, multifocality, and extrathyroidal extension (27). By contrast, the link between TPOAb and thyroid cancer remains uncertain; a study even suggested that high-titer TPOAb could lower the risk (28). Thus, compared with TPOAb, TgAb may better capture the immune dysregulation associated with thyroid cancer and serve as a more stable predictor for risk stratification of thyroid nodules.

An aspect ratio >1 and microcalcifications are two key ultrasound features in our model. When the aspect ratio exceeds 1, the nodule grows perpendicular to the thyroid capsule. Unlike the expansive growth of benign nodules along tissue planes, this growth pattern correlates with tumor cell invasion and fibrosis of surrounding tissue (29). On ultrasound, microcalcifications usually appear as tiny bright spots without posterior shadowing; histologically, they correspond to psammoma bodies. Psammoma bodies were once believed to stem from dystrophic calcification due to rapid tumor growth. However, newer evidence indicates that tumor cells play an active part in their formation—for instance, by secreting bone-related proteins such as osteopontin or assembling calcification precursors inside cells (30). Although microcalcifications are highly suggestive of cancer, they can also be seen in benign conditions like nodular goiter or Hashimoto’s thyroiditis (31).

The C‑TIRADS system considers both as sonographic features suspicious for malignancy, and several studies support their role in distinguishing benign from malignant nodules (32, 33). These two features were selected by LASSO from 19 candidate variables, whereas the C-TIRADS category itself was not included in the selection and served only as a reference for comparison. The retention of the combination of aspect ratio >1 and microcalcifications in our cohort suggests its independent discriminative value beyond C-TIRADS classification.

VEGF helps regulate tumor angiogenesis and is upregulated in many common cancers, such as lung, breast, and colorectal tumors (34, 35). Inside thyroid cancers, a hypoxic microenvironment activates the HIF-1α pathway, which increases VEGF expression, promotes abnormal new vessel formation, and drives tumor progression (36). Some of the VEGF made by tumor cells enters the bloodstream, so measuring serum VEGF roughly reflects how active angiogenesis is.

In our cohort, malignant thyroid nodules showed higher serum VEGF levels than benign ones. A nonlinear test gave a *p* value of 0.135, pointing to a largely linear relationship between VEGF and malignancy. On multivariate analysis, serum VEGF emerged as an independent predictor: each 100 pg/mL increase corresponded to about a 3.8-fold rise in risk (OR = 3.798). This matches the report by Yu et al. (12), who confirmed that serum VEGF levels are significantly elevated in patients with papillary thyroid carcinoma and are closely associated with lymph node metastasis and high-risk tumor profiles. Similarly, Karaca et al. (37) saw serum VEGF fall after thyroid cancer surgery, backing the view that circulating VEGF mainly originates from malignant thyroid tissue.

Serum VEGF is not a thyroid-specific marker and may be elevated in autoimmune thyroiditis (AIT) or other inflammatory conditions, which could confound its association with malignancy. In our cohort, however, the VIF for VEGF was below 2, indicating no significant collinearity with TgAb. Moreover, VEGF remained an independent predictor in the multivariable model that included TgAb (OR = 3.798, 95% CI: 1.763–8.677, *P* = 0.002), suggesting its predictive value extends beyond AIT-mediated effects. Nevertheless, VEGF should be interpreted as part of a multi-marker panel, and its value may be attenuated in AIT populations. Future studies incorporating thyroid-specific angiogenic markers such as Galectin-3 or Ang-2 may further enhance disease specificity.

Beyond its role as a circulating marker, VEGF expression is closely linked to BRAF V600E mutation status through the MAPK/ERK pathway, and together they promote tumor progression. Jo et al. (38) demonstrated that BRAF V600E-mutated papillary thyroid carcinoma tissues exhibited higher VEGF levels than wild-type ones, and VEGF expression correlated with tumor size, extrathyroidal invasion, and TNM stage. This relation also exists in other cancer types. Deng et al. (39) found elevated VEGF and increased vascular endothelial cell density in BRAF V600E-mutated colorectal cancer tissues, reinforcing the connection between BRAF mutations and VEGF upregulation. In an anaplastic thyroid cancer model, Husain et al. (40) reported that vemurafenib reduced VEGFA and VEGFC secretion by tumor cells, thus suppressing tumor-induced angiogenesis. No previous study has combined serum VEGF levels with BRAF V600E mutation status specifically for C-TIRADS 3 and 4 nodules to provide additional biological insight into malignancy risk.

Our model has several advantages over earlier tools. First, it focuses specifically on C-TIRADS 3 and 4 nodules, a notoriously difficult group that presents diagnostic and management challenges. Second, it combines three predictor types: a driver mutation (BRAF V600E), a circulating angiogenic marker (VEGF), and a thyroid autoantibody (TgAb). Together, these elements cover molecular biology, tumor angiogenesis, and immune microenvironment, giving a more complete basis for assessing the malignant behavior of thyroid nodules. Beyond its diagnostic performance, the nomogram may also assist in guiding post-FNAC clinical management. For nodules with a high predicted probability of malignancy on the nomogram, particularly when BRAF V600E is positive and high-risk ultrasound features are present, the model supports timely surgical resection. For nodules with indeterminate cytology (Bethesda III/IV), the nomogram provides a quantitative risk estimate that helps distinguish lesions warranting surgery from those suitable for continued surveillance. For nodules with benign cytology (Bethesda II) but highly suspicious imaging features, a low predicted probability, along with negative BRAF V600E and low VEGF levels, may provide molecular reassurance for active surveillance. By offering a personalized management pathway for different risk levels after FNAC, this model may help optimize the clinical workflow for patients with C-TIRADS 3/4 nodules, reducing unnecessary diagnostic surgeries and treatment delays.

This study has several limitations. For some patients in the benign group, we did not have postoperative histopathological confirmation; instead, we relied on FNAC cytology (Bethesda II) together with 12-month ultrasound follow-up to classify nodules as benign. To address this, we performed a sensitivity analysis restricted to surgically confirmed cases (*n* = 183), which yielded virtually identical performance (AUC 0.897 vs. 0.890), supporting the robustness of our findings. Nevertheless, 12 months of follow-up may be insufficient to detect progression in very indolent malignancies, and longer follow-up or surgical confirmation is needed in future studies.

The sample size, particularly the number of malignant events (76), did not meet the minimum of 135 events recommended by the pmsampsize method based on all 19 candidate predictors. LASSO regression and bootstrap internal validation were employed to mitigate overfitting, with a low optimism bias of 0.004, suggesting acceptable internal stability. External validation in larger cohorts is still essential.

We did not include Bethesda cytological categories as predictors, as the model is intended for scenarios where cytology is indeterminate (Bethesda III/IV) or discordant with imaging. Since the model was trained on a mixed cohort rather than exclusively on these subgroups, its applicability requires prospective validation in enriched cohorts.

Furthermore, this was a single-center retrospective study with inherent selection bias, as eligibility required complete clinical, serological, and BRAF data. All 300 patients in the final cohort had 100% complete data, and excluded patients with retrievable data showed no significant differences in age, sex, or C-TIRADS distribution (all *P* > 0.05). Since exclusion was based on data completeness rather than outcome, this bias is unlikely to be systematic.

Moreover, the cohort had higher malignancy prevalence than the general screening population, and the histological spectrum was predominantly PTC (97.4%), limiting its applicability to general screening populations and rare subtypes.

Finally, serum VEGF is not thyroid-specific and may be elevated in autoimmune thyroiditis or other inflammatory conditions, though collinearity with TgAb was low (VIF < 2). The linearity assumption for VEGF also requires external validation.

Future multicenter prospective studies with larger, more diverse cohorts are needed to confirm the model’s robustness and generalizability, particularly in Bethesda III/IV nodules and rare histological subtypes. Workflow evaluations and cost-effectiveness analyses are also warranted before clinical implementation.

## 5 Conclusion

In summary, the nomogram provides an individualized risk estimate that may assist surgical decision-making between surgery and active surveillance in the post-FNAC setting for C-TIRADS 3/4 nodules. However, because our study adopted a single-center retrospective design, the model’s generalizability requires external validation in multicenter prospective cohorts before clinical implementation, along with workflow evaluations and cost-effectiveness analyses.

## Data Availability

The raw data supporting the conclusions of this article will be made available by the authors, without undue reservation.
