# Supplementary material for: Nomogram integrating BRAF V600E, serum biomarkers, and ultrasound features for malignancy risk stratification in C-TIRADS category 3 and 4 thyroid nodules
Source: Front Med (Lausanne). 2026 Aug 27;13:1908160. doi: 10.3389/fmed.2026.1908160 (PMC13558111; doi:10.3389/fmed.2026.1908160)
Supplement: Supplementary file 1 [file Supplementary_file_1.DOCX]

Supplementary Material

# 1 Supplementary Tables

**Supplementary Table S1.** Interobserver agreement for ultrasound features

| Ultrasound feature | Cohen's Kappa (κ) | 95% CI | P value |
| --- | --- | --- | --- |
| Maximum diameter  (≤1 cm vs. >1 cm) | 0.80 | 0.72-0.88 | <0.001 |
| Aspect ratio  (≤1 vs. >1) | 0.92 | 0.85-0.98 | <0.001 |
| Echogenicity  (Non-hypoechoic vs. Hypoechoic) | 0.85 | 0.78-0.91 | <0.001 |
| Microcalcifications  (No vs. Yes) | 0.86 | 0.79-0.93 | <0.001 |
| Blood flow signal  (No vs. Yes) | 0.88 | 0.81-0.95 | <0.001 |
| Border clarity  (Clear vs. Obscure) | 0.87 | 0.80-0.95 | <0.001 |
| Shape  (Regular vs. Irregular) | 0.90 | 0.83-0.97 | <0.001 |
| Composition  (Non-solid vs. Solid) | 0.91 | 0.85-0.93 | <0.001 |
| Cervical lymph node  (Invisible vs. Visible) | 0.83 | 0.76-0.90 | <0.001 |

Cohen‘s kappa coefficients were calculated based on independent assessments by two senior radiologists (each with >10 years of experience). Disagreements were resolved by a third senior sonographer (≥15 years of experience).

**Supplementary Table S2.** Coding of candidate variables for LASSO regression analysis

| Variable name | Type | Coding / values |
| --- | --- | --- |
| Age | Continuous | years |
| Sex | Binary | 0 = female, 1 = male |
| BMI | Continuous | kg/m² |
| BRAF V600E mutation | Binary | 0 = wild‑type, 1 = mutated |
| TgAb | Binary | 0 = negative, 1 = positive |
| TPOAb | Binary | 0 = negative, 1 = positive |
| VEGF | Continuous | pg/mL (divided by 100 for analysis) |
| NLR | Continuous | ratio |
| PLR | Continuous | ratio |
| MLR | Continuous | ratio |
| Maximum diameter | Binary | 0 = ≤1 cm, 1 = >1 cm |
| Aspect ratio | Binary | 0 = ≤1, 1 = >1 |
| Echogenicity | Binary | 0 = non‑hypoechoic, 1 = hypoechoic |
| Microcalcifications | Binary | 0 = no, 1 = yes |
| Blood flow signal | Binary | 0 = no, 1 = yes |
| Border clarity | Binary | 0 = clear, 1 = obscure |
| Shape | Binary | 0 = regular, 1 = irregular |
| Composition | Binary | 0 = non‑solid, 1 = solid |
| Cervical lymph node | Binary | 0 = invisible, 1 = visible |

All continuous variables were used as raw values; binary variables were coded as 0/1. VEGF was divided by 100 before entry into the model to improve clinical interpretability.

**Supplementary Table S3.** Variance inflation factor (VIF) for the five selected predictors

| Predictor | VIF |
| --- | --- |
| BRAF V600E mutation | 1.112 |
| VEGF (per 100 pg/mL) | 1.039 |
| TgAb positivity | 1.187 |
| Aspect ratio >1 | 1.056 |
| Microcalcifications | 1.100 |

All VIF values below the conventional threshold of 5, indicating no problematic multicollinearity. VIFs derived from the standard logistic regression model.

# 2 Supplementary Figures

**Supplementary Figure 1.** Restricted cubic spline for serum VEGF and malignancy risk.

We used a restricted cubic spline with three knots at the 10th, 50th, and 90th percentiles. The solid line shows the predicted probability of malignancy as a function of serum VEGF (per 100 pg/mL); the shaded area is the 95% confidence interval. The overall association was significant (χ² = 20.89, P < 0.001), while the nonlinear component was not (P = 0.135), supporting a linear relationship.
